# Highly Sensitive and Specific Detection of Influenza A Viruses Using Bimolecular Fluorescence Complementation (BiFC) Reporter System

**DOI:** 10.3390/bios13080782

**Published:** 2023-08-02

**Authors:** Ui Jin Lee, Yunkwang Oh, Oh Seok Kwon, Yong-Beom Shin, Moonil Kim

**Affiliations:** 1 Critical Diseases Diagnostics Convergence Research Center, Korea Research Institute of Bioscience and Biotechnology (KRIBB), 125 Gwahang-ro, Yuseong-gu, Daejeon 34141, Republic of Korea; waainie@eyebiokorea.com (U.J.L.); oyk0213@kribb.re.kr (Y.O.); 2 SKKU Advanced Institute of Nanotechnology (SAINT), Sungkyunkwan University, Suwon 16419, Republic of Korea; oskwon79@skku.edu; 3 Department of Nano Science and Technology, Sungkyunkwan University, Suwon 16419, Republic of Korea; 4 Department of Nano Engineering, Sungkyunkwan University, Suwon 16419, Republic of Korea; 5 Bionanotechnology Research Center, Korea Research Institute of Bioscience and Biotechnology (KRIBB), Daejeon 34141, Republic of Korea; ybshin@kribb.re.kr; 6 BioNano Health Guard Research Center (H-GUARD), Daejeon 34141, Republic of Korea; 7 KRIBB School, Korea University of Science and Technology (UST), 217 Gajeong-ro, Yuseong-gu, Daejeon 34113, Republic of Korea

**Keywords:** bimolecular fluorescence complementation, BiFC, influenza A virus, IAV, biosensor

## Abstract

In this study, we developed a highly sensitive and specific bimolecular fluorescence complementation (BiFC)-based influenza A virus (IAV)-sensing system by combining a galactose/glucose-binding protein (GGBP) with an N-terminal large domain (YN1-172) and a C-terminal small domain (YC173-239) made up of enhanced yellow fluorescence protein (eYFP). The GGBP-based BiFC reporter exhibits the fluorescence reconstitution as a result of conformational changes in GGBP when lactose, which was derived from 6′-silalyllactose and used as a substrate for neuraminidase (NA), binds to GGBP in the presence of IAV. The system showed a linear dynamic range extending from 1 × 10^0^ to 1 × 10^7^ TCID_50_/mL, and it had a detection limit of 1.1 × 10^0^ TCID_50_/mL for IAV (H1N1), demonstrating ultra-high sensitivity. Our system exhibited fluorescence intensity enhancements in the presence of IAV, while it displayed weak fluorescence signals when exposed to NA-deficient viruses, such as RSV A, RSV B, adenovirus and rhinovirus, thereby indicating selective responses for IAV detection. Overall, our system provides a simple, highly sensitive and specific IAV detection platform based on BiFC that is capable of detecting ligand-induced protein conformational changes, obviating the need for virus culture or RNA extraction processes.

## 1. Introduction

Influenza A virus (IAV) is the predominant type responsible for most cases of influenza, and it annually causes widespread worldwide outbreaks [1,2]. The influenza virus encompasses diverse lineages that contribute to the periodic occurrence of seasonal influenza. For example, the H1N1 lineage was the causative agent of the global pandemic that occurred from 2009 to 2010 [3,4]. Neuraminidase (NA), which is one of the major surface proteins of IAV, plays a crucial role in the release of nascent viral particles assembled in infected cells. It facilitates the discharge of these particles from cells by cleaving sialic acid residues found in the glycan component attached to glycoproteins on the cell’s plasma membrane [5,6,7].

The diagnosis of human IAV infections is typically achieved through viral culture, molecular-based diagnosis tests and immunological diagnostic approaches [8,9,10,11]. In conventional practice, the measurement of virus infectivity involves conducting plaque or focus assays in cell cultures [12,13]. These methods continue to be widely used to assess virus infectivity due to their reliability. Nevertheless, they require several days to obtain results and ensure the proper handling of the organism [14]. Molecular-based diagnosis tests encompass techniques such as the reverse transcription PCR (RT-PCR), real-time PCR and isothermal amplification methods. PCR has the high sensitivity required to detect low virus quantities during the early stages of infection. However, it requires specialized equipment and trained personnel and may not provide rapid results [15,16]. Isothermal amplification methods, like nucleic acid sequence-based amplification (NASBA), loop-mediated isothermal amplification (LAMP), recombinase polymerase amplification (RPA), etc., offer relatively fast and sensitive reaction, thus becoming gold-standard amplification techniques. Despite the advantages of isothermal reaction, the low temperature between 30 and 55 °C makes it prone to unspecific primer binding that might cause unspecific amplifications [17,18]. Immunological diagnostic approaches, such as the enzyme-linked immunosorbent assay (ELISA), immunofluorescence assay (IFA) and rapid influenza diagnostic test (RIDT), are widely used to perform IAV detection [19,20,21]. These methods, along with standardized protocols and commercial kits, enable rapid detection of IAV in laboratory and field settings. However, they have lower sensitivity than molecular diagnostics and may exhibit cross-reactivity with other viral antigens [22].

A bimolecular fluorescence complementation (BiFC) system involves the expression of fluorescence proteins as two fragments, which do not produce fluorescence when far apart, but restore fluorescence when brought close together [23,24,25]. The system has been widely regarded as a potent tool that utilizes a split reporter strategy to analyze protein conformational changes, as well as protein–protein interactions, in various cell types and organisms [26,27]. In this study, we developed an IAV sensing system by combining a GGBP with a BiFC reporter protein and using 6′-sialyllactose as a substrate. To the best of our knowledge, our research represents the first application of the BiFC system used to perform virus detection, confirming its potential applicability. The designed molecular system utilizes a split eYFP as a transducer to convert the structural changes of GGBP into fluorescence. The underlying principle of the GGBP-based BiFC reporter system relies on the fluorescence reconstitution triggered by the conformational changes in GGBP upon the binding of lactose, which is cleaved from 6′-sialyllactose, to GGBP in the presence of IAV. Our system demonstrated outstanding sensitivity, wide dynamic range and specificity towards IAV. In particular, the achieved detection limit of 1.1 × 10^0^ TCID_50_/mL for IAV (H1N1) obtained in the current study demonstrates the potential applicability of our system to on-site testing or primary screening, as highly sensitive and direct detection without virus amplification is required.

## 2. Materials and Methods

### 2.1. Materials, Strains, Vectors and Enzymes

Glucose, galactose and lactose were purchased from Sigma-Aldrich (St. Louis, MO, USA), and 6′-sialyllactose was obtained from Biosynth (Compton, UK). Oseltamivir carboxylate, which is the active form of oseltamivir, was obtained from Adamas Pharmaceuticals (Emeryville, CA, USA). *E. coli* strian DH5α was used as a host for subcloning, and *E. coli* BL21 (DE3) (Novagen, Madison, WI, USA) was used for gene expression. pGEM-T (Promega, Madison, WI, USA) and pET-21a(+) (Novagen, Madison, WI, USA) were used as vectors for subcloning and protein expression, respectively. All of the restriction enzymes and modifying enzymes were purchased from Nanohelix (Daejeon, Korea), and they were used according to the recommendations of the supplier. A preparation of vector DNA was carried out using a QIAEX II gel extraction kit (Qiagen, Hilden, Germany).

### 2.2. Viruses

Influenza A viruses (H1N1, H1N2, H3N8 and H6N5) suspended in cell culture medium (MEM, MDCK cells) were obtained from the BioNano Health Guard Research Center (H-GUARD). Respiratory syncytial virus (RSV), adenovirus and rhinovirus were provided by the Korea Bank for Pathogenic Viruses (KBPV).

### 2.3. Reporter Plasmid Construction

YN1YC173 and YN1YC155 plasmids were constructed using the multiple fragment assembly procedure [28]. The detailed reporter plasmid construction method, including all primers used, is described in the Supplementary Materials Information (Materials and Methods S1).

### 2.4. Site-Directed Mutagenesis

To enable the cloning of the mutant YN1YC173-Asp14Ile, overlap extension PCR-based site-directed mutagenesis was conducted using flanking primers (forward primer: 5′-CAT ATG GTG AGC AAG GGC GAG GAG-3′; reverse primer: 5′-CTC GAG TTT CTT GCT GAA TTC AGC-3′) on both ends of the template, as well as internal primers (forward primer: 5′-TAT AAG TAC GAC ATC AAC TTT ATG TCT-3′; reverse primer: 5′-AGA CAT AAA GTT GAT GTC GTA CTT ATA-3′) that contain the base changes in interest and bind to the region where the replacement will occur. The resulting PCR products were then inserted into the pET-21a(+) plasmid at the NdeI/XhoI sites.

### 2.5. Protein Expression and Purification

For the recombinant protein expression and purification, plasmids were transferred to the expression host, namely *E. coli* BL21 (DE3), and plated on Luria–Bertani (LB) plates. A single colony derived from a fresh plate was picked and grown at 37 °C in 3 mL of LB broth, which contained 100 mg/mL of ampicillin until OD_600_ = 0.6. They were then inoculated in 100 mL of LB with ampicillin. Cells were grown at 37 °C via shaking until OD_600_ = 0.6. Cells were induced with 1 mM of isopropyl-2-D-thiogalactopyranoside (IPTG) (Gibco BRL, Grand Island, NY, USA) and grown for 4 h. Cells were then harvested via centrifugation at 6000 g at 4 °C for 10 min. Harvested cells were resuspended in 50 mM of Tris-HCl buffer (pH 8.0) and disrupted via sonication. The crude cell lysates were separated into total, soluble and insoluble fractions, which were analyzed using 10% SDS-PAGE. In order to purify the recombinant proteins, 10 mL of the crude cell lysates were loaded onto an IDA-miniexcellose affinity column (Keyprogen, Daejeon, Korea). The recombinant proteins were subsequently eluted with 5 mL of 0.5 M imidazole in the same buffer (50 mM phosphate, 0.5 N NaCl, pH 8.0). The purified proteins were then dialyzed against phosphate-buffered saline (PBS, pH 7.4) overnight at 4 °C. The dialyzed proteins were further purified via size exclusion chromatography (SEC) using a Superdex G75 column (GE Healthcare, Salt Lake City, UT, USA). The protein concentration was determined via Nano drop (Thermo scientific, Waltham, MA, USA). Finally, all recombinant proteins were concentrated to 1 mg/mL and stored in −80 °C for further experiments.

### 2.6. Fluorescence Measurement

The BiFC reporter protein and 6′-sialyllactose were utilized as components of the reporter system for the IAV assay. The concentration of the reporter protein used was 10 ug/mL, which was determined based on our preliminary studies as being the minimum concentration at which fluorescence was maximally reconstituted in the presence of 10 mM lactose, which is a concentration sufficient to induce conformational changes in GGBP (data not shown). Based on the maximum fluorescence enhancement observed with 10 mM lactose in response to 10 ug/mL reporter protein, the 6′-sialyllactose concentration was 10 mM in all experiments based on BiFC complex formation. According to previous reports, there is a delay between the time at which conformational changes occur and the time at which the complex becomes fluorescent, which occurs due to the slow rate of the chemical reactions required to produce the fluorophore [29]. In the current work, an incubation time of 1 h was chosen for the mixture of the reporter protein, 6′-sialyllactose and IAV, which was based on the comparative test results regarding fluorescence enhancement at various incubation times (data not shown). For the measurement of sensitivity in IAV detection, the mixture of 10 ug/mL of reporter protein and 10 mM of 6′-sialyllactose was incubated at 37 °C for 1 h, which had various concentrations of IAV ranging from 1 *×* 10^0^ to 1 *×* 10^7^ TCID_50_/mL. The relative fluorescence intensity was measured as the fluorescence reconstitution using a Tecan Infinite 200 microplate reader (Tecan, Männedorf, Switzerland) at excitation/emission wavelengths of 513/530 nm (excitation/emission slit widths 5/5 nm). After background subtraction, the fluorescence signal was presented as the mean ± SEM in the average relative fluorescence units (RFU). All fluorescence measurements reported here were repeated in a minimum of three independent experiments, and the results of three experiments were averaged.

## 3. Results and Discussion

### 3.1. Principle of Detection

**Figure 1:**
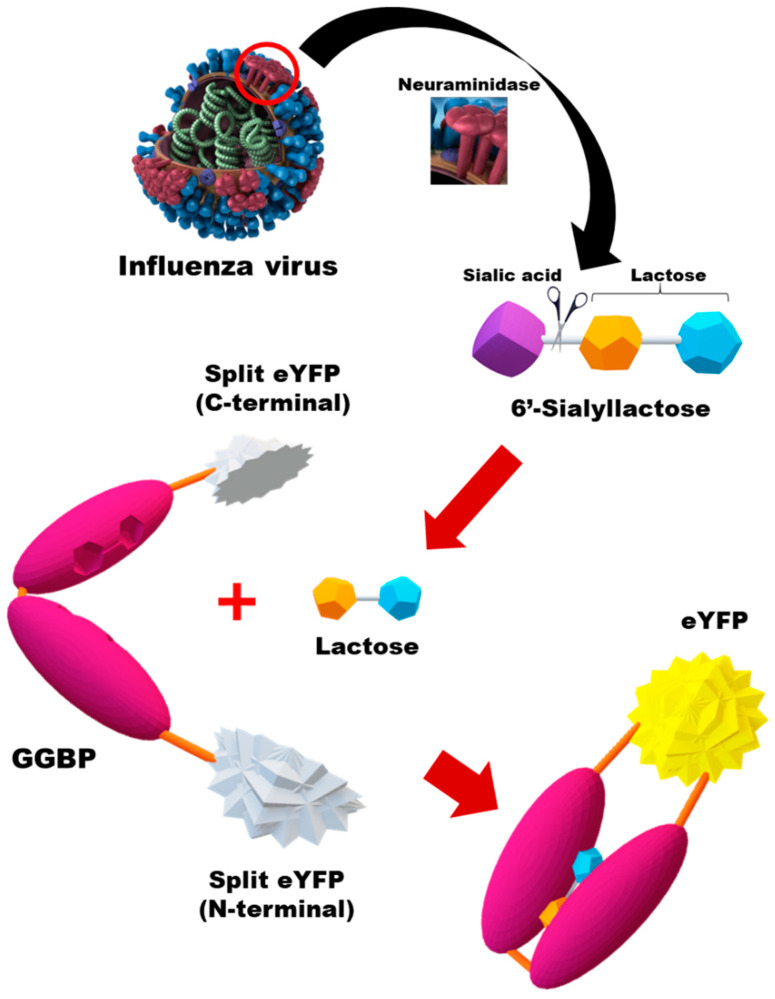
Schematic diagram of in vitro detection of IAV (H1N1) using the GGBP-based BiFC reporter system. When the NA of IAV cleaves the sialic acid residues of 6′-sialyllactose, lactose is released. Upon the binding of the released lactose to the binding pocket between the two lobes of GGBP, structural changes are induced in GGBP, leading to the reconstitution of split eYFP fragments. Red empty circle indicates NA.

GGBP, which is a periplasmic binding protein found in bacteria, undergoes conformational changes upon binding to sugar ligands (i.e., galactose, glucose, lactose, etc.) [30,31,32]. This structural alteration has been previously utilized for the identification of galactose and glucose ligand binding by introducing fluorescent tags into the GGBP [33,34]. When integrated with the BiFC system, the unique hinge–twist motion of the GGBP can be harnessed for biosensing applications. Herein, we aimed to develop an IAV sensing system designed by combining GGBP and the BiFC reporter protein. Figure 1 shows a schematic diagram of the in vitro detection of IAV using a GGBP-based BiFC reporter system. In brief, the NA activity of IAV cleaves the sialic acid moiety from 6′-sialyllactose, releasing lactose in a free state. Upon the binding of the released lactose to the binding site between two lobes of GGBP, the proximity between the lobes is induced via hydrogen bonding between the lobe and the sugar ligand, resulting in a closed configuration. In our study, we employed the EYFP fluorescence complementation system, which was initially developed by Hu et al. [33] in 2002. Fluorescence reconstitution occurs due to ligand-induced structural changes in GGBP, and the fluorescence intensity directly correlates with the concentration of IAV, allowing quantitative measurement to occur.

### 3.2. Characterization of the GGBP-Based BiFC Reporter System

**Figure 2:**
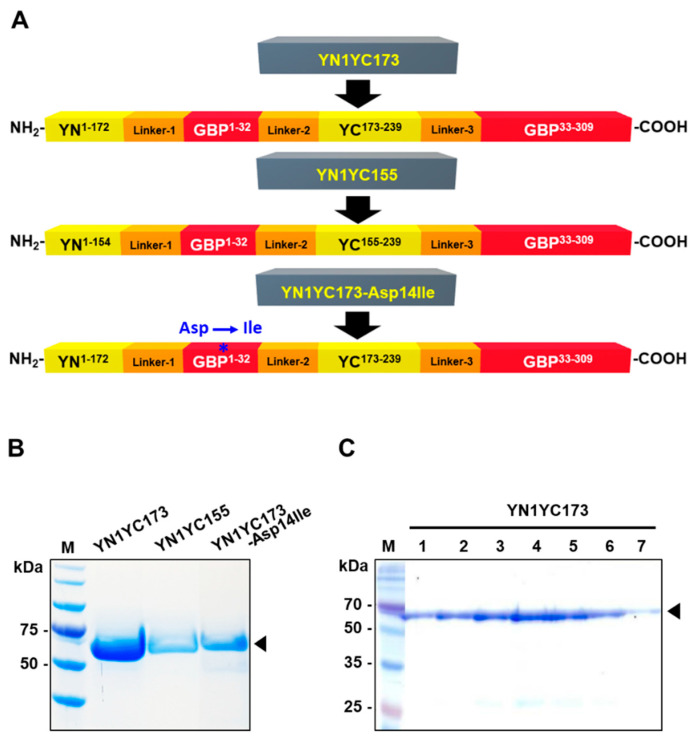
Construction, expression and purification of the reporter proteins. (**A**) Construction of the GGBP-based BiFC reporters. The YN1YC173 and YN1YC155 reporters comprise 4 domains of YN1-172-linker-1, GGBP1-32-linker-2, YC173-239-linker-3 and GGBP33-309, and YN1-154-linker-1, GGBP1-32-linker-2, YC155-239-linker-3 and GGBP33-309, respectively. The YN1YC173-Asp14Ile reporter is a mutant construct of YN1YC173, in which Asp-14 (*) of GGBP is replaced with an aliphatic residue (Ile). Linker-1, GTSSHM; Linker-2, GTGSGS; Linker-3, GSGHM. (**B**) SDS-PAGE analysis of the recombinant GGBP-based BiFC reporters (YN1YC173, YN1YC155 and YN1YC173-Asp14Ile). After IPTG induction, purified reporter proteins were analyzed using 10% SDS–PAGE gel. Lane 1, protein marker; Lane 2, YN1YC173; Lane 3, YN1YC155; Lane 4, YN1YC173-Asp14Ile. (**C**) Analysis of the protein-containing fractions that contain SDS-PAGE. The numbers in the lanes correspond to the fractions of the eluted YN1YC173 reporter protein. The arrowhead indicates the expressed 63.8-kilodalton reporter proteins.

Figure 2A shows construction of the GGBP-based BiFC reporter system. Based on previous studies regarding the split positions of eYFP, two types of reporter variants were generated, namely YN1YC155, which harbored the split between the seventh and eighth β-strands (the cut site between residues 154 and 155) [35,36], and YN1YC173, which harbored the split between the eighth and ninth β-strands (the cut site between residues 172 and 173) [37,38] of eYFP. The two non-fluorescent fragments of eYFP were located at the N-terminus of GGBP and the loop region between residues 32 and 33 of GGBP, and they did not incorporate any amino acid residues that contributed to ligand recognition, as previously reported in [30]. The construction and purification of the reporter proteins were described in detail in the Section 2. After IPTG induction, there was an obvious band around the molecular weight of 63.8 kDa, which was consistent with the expected molecular weight of the recombinant reporter proteins (Figure 2B); the YN1YC173 reporter protein profiles of the different fractions derived from the purification are shown in Figure 2C.

**Figure 3:**
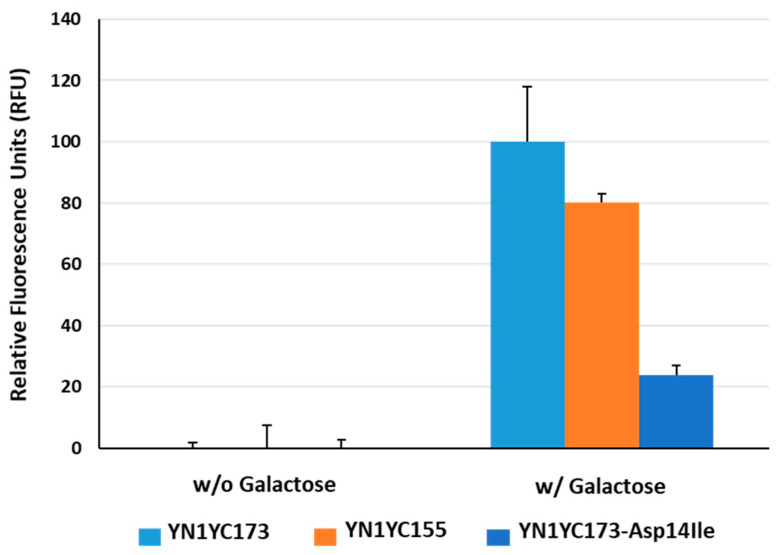
Functional evaluation of the GGBP-based BiFC reporter system. The fluorescence reconstitution in response to a sugar ligand was compared among YN1YC173, YN1YC155 and YN1YC173-Asp14Ile.

Figure 3 illustrates the comparison between fluorescence intensity enhancements in response to a sugar ligand (i.e., galactose) that occur between the two reporters. The average relative fluorescence units (RFU) in the YN1YC155 reporter were observed to be lower than those in the YN1YC173 reporter, having approximately 80% of the value of the YN1YC173 reporter. Based on the responses of the reporter proteins to the sugar ligand, it was concluded that among the two available reporters, the YN1YC173 reporter is more suitable for use as an IAV sensor. Therefore, we decided to utilize the YN1YC173 reporter for further experiments related to IAV detection.

Asp-14 in GGBP is situated at the ligand-binding site and forms hydrogen bonds with the hydroxyl epimers at position 4 of glucose and galactose [39]. Therefore, substituting Asp with an alternative residue was expected to alter the specificity of GGBP. To examine this assumption’s validity, a mutant BiFC reporter YN1YC173-Asp14Ile was prepared through site-directed mutagenesis of GGBP, in which Asp-14 was replaced with an aliphatic residue (Ile). As shown in Figure 3, the fluorescence recovery of the mutant reporter was observed at a level of 24% compared to that of the wild reporter (YN1YC173) in response to the sugar ligand. Given that the increase in the fluorescence intensity of the reporter reflects conformational changes in GGBP in the presence of its cognate ligand, this result suggests that although the sugar ligand has the ability to bind to the wild reporter, its ability to bind to the mutant reporter is limited, which aligns with the observations reported by Sakaguchi-Mikami et al., who stated that the Kd value of the mutant GGBP (Asp14Ile) for galactose was found to be 35 μM, whereas that of the wild GGBP was found to be 0.25 μM, thereby indicating that the mutant GGBP (Asp14Ile) lost its galactose-binding ability [39]. It is noteworthy that in the absence of galactose, the average RFU in the wild reporter sample was almost negligible, whereas in the presence of galactose, it was significantly lower than that found in the mutant reporter samples. This result may imply that the design of the IAV sensing reporter incorporates a structure that prevents the unintended self-assembly of split eYFP fragments in the absence of a sugar ligand.

**Figure 4:**
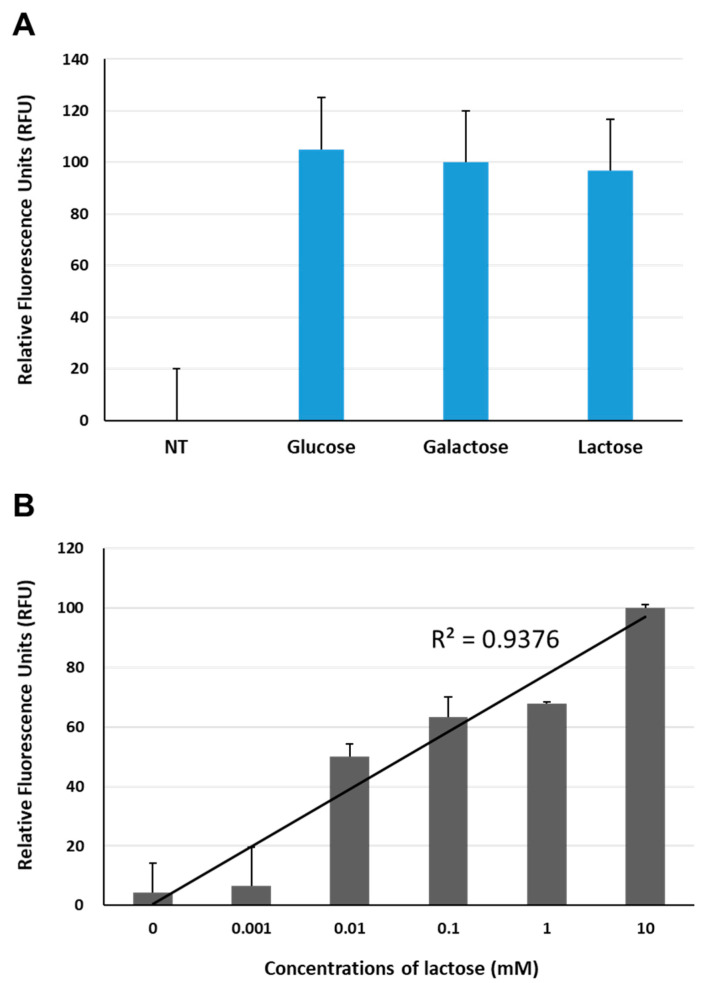
Measurement of fluorescence complementation of YN1YC173 (**A**) in response to glucose (10 mM), galactose (10 mM) and lactose (10 mM), as well as (**B**) at various concentrations of lactose. NT, non-treatment.

NA serves as a potential enzymatic marker for rapid viral diagnosis. Recently, an intriguing study of the detection of IAV through the enzymatic activity of NA using a nanopore sensor has been reported [40]. In that study, 6′-sialylgalactose was employed as a substrate for viral NA, and the structural changes in GGBP, which were induced by galactose cleaved from 6′-sialylgalactose, were measured using a Cytolysin A-based nanopore sensor. In general, in GGBP-based IAV sensors that target NA, either 6′-sialylgalactose or 6′-sialylglucose can be used as substrates for NA. From an economic point of view, choosing 6′-sialyllactose as a substrate that interacts with NA can be beneficial due to its cost effectiveness. Since it is a naturally occurring oligosaccharide that is abundant in mammalian milk, it can be sourced from a readily available and abundant resource. Moreover, lactose is not typically present in human saliva or nasal fluids; hence, the exclusion of interference issues caused by lactose in the samples can support the choice of 6′-sialyllactose. Therefore, to explore the potential use of 6′-sialyllactose as a substrate for the BiFC-based IAV sensor, we compared the fluorescence reconstitutions of the reporter in response to glucose (10 mM), galactose (10 mM) and lactose. As shown in Figure 4A, upon comparison with the fluorescence intensity of the reporter without any ligand, three all ligands clearly induced fluorescence restoration with similar fluorescence enhancements under our experimental settings. These findings are in good agreement with the results reported by Taneoka et al., who stated that GGBP exhibits similar levels of reactivity with not only glucose and galactose, but also lactose [30]. Next, to verify whether the fluorescence recovery of the BiFC reporter occurred due to specific interaction with lactose, we measured the changes in the fluorescence complementation of the BiFC reporter in response to lactose concentrations that ranged from 1 to 10 mM. As shown in Figure 4B, the BiFC reporter exhibited fluorescence intensity enhancements with increasing lactose concentration, displaying a high degree of regression analysis that fits with the presence of a high R-squared value (R^2^ = 0.9376, *p* < 0.01), thereby indicating that lactose has the ability to induce conformational alterations in the reporter protein.

### 3.3. Functional Evaluation for IAV Detection

**Figure 5:**
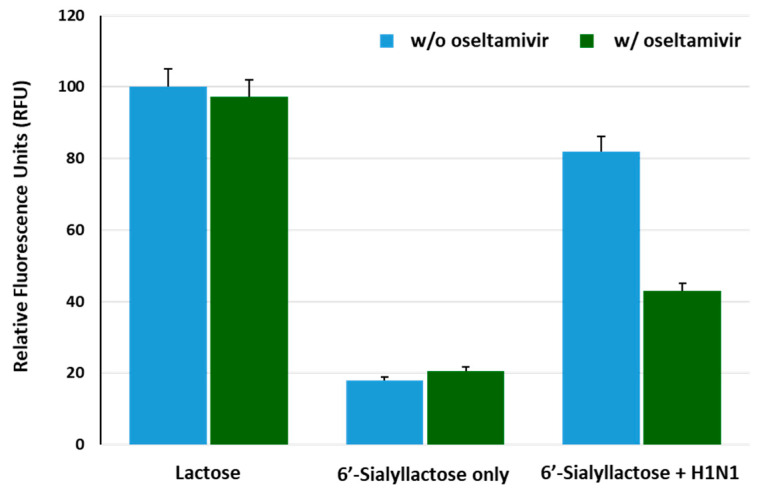
Functional evaluation of the GGBP-BiFC-based IAV sensor. The fluorescence intensity enhancements of the reporter (10 ug/mL) were analyzed in response to lactose (10 mM), 6′-sialyllactose (10 mM) only and 6′-sialyllactose treated with H1N1 (4 *×* 10^3^ TCID_50_/mL). To examine the reliability of the H1N1 detection system, the inhibitory effect of the oseltamivir (10 μM) on NA activity was tested.

To assess the efficacy of our reporter system regarding the detection of IAV (H1N1), we investigated whether lactose was freely released from 6′-sialyllactose substrate in response to IAV, subsequently inducing fluorescence reconstitution. The BiFC response of the reporter protein was analyzed upon treatment with lactose (10 mM), 6′-sialyllactose (10 mM) only and 6′-sialyllactose with H1N1 (4 *×* 10^3^ TCID_50_/mL). Additionally, the influence of oseltamivir (10 μM), which is an inhibitor of NA, on the fluorescence recovery changes was investigated for each sample. As shown in Figure 5, an increase in the fluorescence intensity was observed in reporter samples treated with lactose, while oseltamivir did not affect the fluorescence signal. In reporter samples treated with 6′-sialyllactose only, the fluorescence restoration decreased to approximately 20% of that observed in lactose-treated reporter samples, regardless of oseltamivir treatment, indicating that fluorescence reconstitution did not occur unless lactose was freely released via cleavage of sialic acid residues from 6′-sialyllactose. However, in samples that contained both 6′-sialyllactose and H1N1, approximately 85% efficiency of fluorescence recovery was observed in the absence of oseltamivir, whereas the presence of oseltamivir led to a decrease in fluorescence intensity of nearly half of its level. This result can be interpreted as the inhibitory effect of oseltamivir on the NA activity of H1N1, which hinders the detachment of lactose from 6′-sialyllactose, thus preventing the structural changes in GGBP and resulting in the inhibition of the fluorescence reconstitution.

### 3.4. Sensitivity for IAV Detection

**Figure 6:**
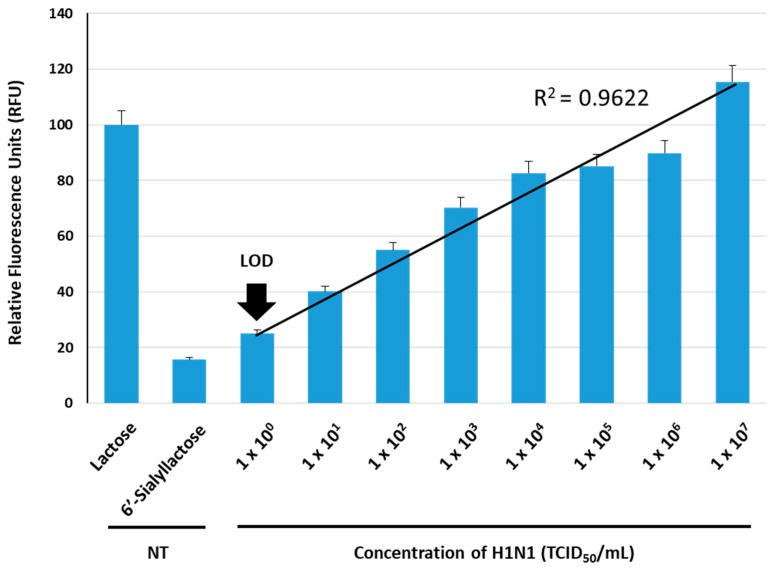
Sensitivity of the GGBP-based BiFC reporter system to IAV detection. The average RFU under various concentrations of H1N1 that ranged from 1 *×* 10^0^ to 1 *×* 10^7^ TCID_50_/mL were measured. NT, non-treatment.

We examined the IAV (H1N1) concentration-dependent fluorescence reconstitutions derived from the BiFC reporter. The H1N1 samples were prepared at concentrations ranging from 1 *×* 10^0^ to 1 *×* 10^7^ TCID_50_/mL via 1/10 serial dilution, with conditions of 10 ug/mL reporter protein and 10 mM 6′-sialyllactose used. The 6′-sialyllactose sample in the absence of virus (NT, non-treatment) was prepared as a control. The fluorescence intensity in response to 10 mM of lactose was set at 100.0%, and the impact of virus concentration variations on fluorescence reconstitution was monitored. Virus amplification was performed by culturing MDCK cells in DMEM medium that contained trypsin (1 ug/mL), followed by virus treatment and harvesting of samples after 3–5 days of cell destruction. The BiFC reporter protein, 6′-sialyllactose and H1N1 were placed in each single well of a 96-well plate and incubated at 37 °C for 1 h, allowing sufficient time for the viral NA to liberate sialic acid residues from 6′-sialyllactose and the released lactose to induce structural changes in GGBP. Figure 6 shows the correlation between H1N1 concentration and fluorescence recovery. The average RFU gradually increased as the virus concentration increased, displaying a fitted line graph with a high R-squared value (R^2^ = 0.973, *p* < 0.01). Our system exhibited a linear dependency within the range of from 1 *×* 10^0^ to 1 *×* 10^7^ TCID_50_/mL, allowing the quantitative measurement of H1N1. The estimated limit of detection (LOD) of this method was 1.1 *×* 10^0^ TCID_50_/mL, a value that was calculated as LOD = background signal + 3∙SD (background signal). Narrow variations in fluorescence signals were also observed at each point of virus detection, which implies that an increase in the reliable fluorescence signal occurred due to the fluorescence reconstitution triggered as a result of conformational changes in GGBP when lactose bound to GGBP in the presence of H1N1. Typically, the viral concentration of respiratory viruses, including IAV, which is found in droplets released into the air during the initial stages of coughing in infected individuals, is estimated to be approximately 1 *×* 10^3^ TCID_50_/mL. This result means that the limit of detection should be lower than 1 *×* 10^3^ TCID_50_/mL to confirm the presence of infection in the early stages. Over the past few decades, various detection techniques for IAV have been developed to enhance sensitivity. Previously reported PCR-based studies demonstrated a detection limit at the level of 1 *×* 10^2^ TCID_50_/mL [41,42,43]. Previously reported immunodiagnostic assays showed a detection limit that ranged from 1 × 10^3^ to 1 × 10^4^ TCID_50_/mL [44,45,46]. Therefore, when comparing the sensitivity values to those previously reported, our BiFC reporter system exhibited a lower detection limit and a wider dynamic range. In on-site testing situations in which direct detection without the need for virus amplification is required, an ultra-high sensitive sensor is inevitable. In this regard, the achievement of a 1.1 *×* 10^0^ TCID_50_/mL detection limit for H1N1 in the current study indicates that this method holds the potential for application in the field of primary screening, which requires high sensitivity to enable direct detection.

### 3.5. Specificity for IAV Detection

**Figure 7:**
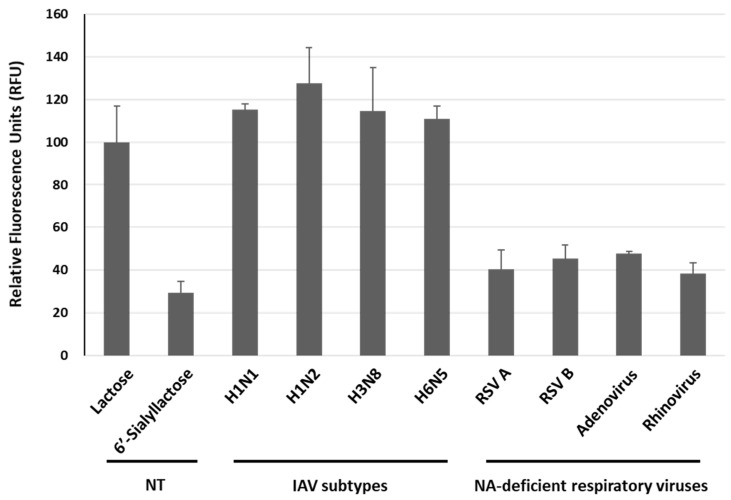
Specificity of the GGBP-based BiFC reporter system regarding IAV detection over other NA-deficient respiratory viruses, such as RSV A, RSV B, adenovirus and rhinovirus. The concentration was 1 *×* 10^7^ TCID_50_/mL for all viruses examined. NT, non-treatment.

To evaluate the specificity of this approach, we investigated four subtypes of IAV (H1N1, H1N2, H3N8 and H6N5) and four respiratory viruses (RSV A, RSV B, adenovirus and rhinovirus). The concentration of each examined viruses was 1 *×* 10^7^ TCID_50_/mL. As shown in Figure 7, the average RFUs induced by H1N1, H1N2, H3N8 and H6N5 were 115.2%, 127.5%, 114.7% and 110.9%, respectively, with the signal value for the lactose sample in the absence of virus (NT, non-treatment) set at 100.0%. Given that the current system relies on the activity of NA within IAV, it was an easily predictable result. In contrast, the average RFU in response to other respiratory viruses, such as RSV A, RSV B, adenovirus and rhinovirus, were measured at 40.5%, 45.4%, 47.7% and 38.3%, respectively, exhibiting weak signals in the presence of these NA-deficient viruses, thus indicating the specificity of our system required for IAV detection. Therefore, we expect that the GGBP-based BiFC reporter system would be useful for the specific detection of IAV.

## 4. Conclusions

In summary, a facile, highly sensitive and specific IAV sensing system, which was designed by combining a GGBP and a BiFC reporter protein while using 6′-sialyllactose as a substrate, was developed. The fluorescence reconstitution of the GGBP-based BiFC reporter was triggered via conformational changes in GGBP upon the binding of lactose, which was cleaved from 6′-silalyllactose via NA enzyme activity of IAV, to the GGBP. The BiFC reporter system enables the facile detection of IAV without the need for virus culture or RNA extraction processes. The current system demonstrated exceptional sensitivity in terms of detecting IAV (H1N1) by exhibiting a linear dynamic range that ranged from 1 *×* 10^0^ to 1 *×* 10^7^ TCID_50_/mL, having a remarkable detection limit of 1.1 *×* 10^0^ TCID_50_/mL. The reporter system also exhibited weak fluorescence intensity enhancements in the presence of NA-deficient viruses, indicating the specificity of the system regarding IAV detection. In on-site testing in which immediate virus detection without the requirement of virus amplification is crucial, the demand for an ultra-high sensitive sensor becomes imperative. In this context, our current study’s attainment of a detection limit of 1.1 *×* 10^0^ TCID_50_/mL for H1N1 highlights the potential applicability of our system in the field of primary screening, in which high sensitivity detection and large-scale sample monitoring are required.

## Data Availability

The data presented in this study are available on request from the corresponding author (M.K.).

## Supplementary Materials

The following supporting information can be downloaded via the below link: https://www.mdpi.com/article/10.3390/bios13080782/s1, Materials and Methods S1: Supplementary Materials.
