# Supplementary material for: Highly Sensitive and Specific Detection of Influenza A Viruses Using Bimolecular Fluorescence Complementation (BiFC) Reporter System
Source: Biosensors (Basel). 2023 Aug 2;13(8):782. doi: 10.3390/bios13080782 (PMC10452828; doi:10.3390/bios13080782)
Supplement: Supplementary file 1 [file biosensors-13-00782-s001.zip › biosensors-2497643-supplementary.pdf]

Supplementary Materials

Reporter plasmid construction

The cloning of the YN1YC173 construct was achieved using the multiple fragment assembly method. For the YN1-172-linker-1 fragment, the partial gene that encompassed amino acids 1–172 of eYFP was amplified using the corresponding primers (forward primer: 5′-CAT ATG GTG AGC AAG GGC GAG GAG-3′; reverse primer: 5′-GGA TCC CAT ATG TGA TGA GGT ACC GAT GTT GTG GCG GAT CTT-3′), ensuring the inclusion of the linker-1 sequence (GTSSHM) and specific restriction enzyme cleavage sites (NdeI and BamHI) at the 5' and 3' termini. Similarly, the GGBP1-32-linker-2 fragment involved amplification of the gene segment that encoded amino acids 1–32 of GGBP, along with appropriate primers (forward primer: 5′-GGA TCC GCT GAT ACT CGC ATT GGT-3′; reverse primer: 5′-GTC GAC TGA ACC TGA ACC GGT ACC TGG CGC GGC TTT CGC ATC-3′) that were designed to incorporate the linker-2 sequence (GTGSGS) and desired restriction enzyme cleavage sites (BamHI and SalI). For the YC173-239-linker-3 fragment, the partial gene encoding amino acids 173–239 of eYFP were PCR-amplified using specific primers (forward primer: 5′-GTC GAC GAG GAC GGC AGC GTG CAG-3′; reverse primer: 5′-AAG CTT CAT ATG ACC TGA ACC CTT GTA CAG CTC GTC CAT-3′), which were also designed to include the linker-3 sequence (GSGHM) and desired restriction enzyme cleavage sites (SalI and HindIII). Finally, the GGBP33-309 fragment was generated by amplifying the partial gene encoding amino acids 33–309 of GGBP, utilizing the designated primers (forward primer: 5′-AAG CTT GAT GTT CAG CTG CTG ATG-3′; reverse primer: 5′-CTC GAG TTT CTT GCT GAA TTC AGC-3′) that incorporated the specified restriction enzyme cleavage sites (HindIII and XhoI) at the termini. The plasmid construct design involved combining incompatible-ended fragments and a linearized vector, resulting in the successful construction of the YN1YC173 construct. This result was achieved by mixing the four PCR fragments (YN1-172-linker-1, GGBP1-32-linker-2, YC173-239-linker-3 and GGBP33-309) with the linearized vector (pET-21a(+)), which was cleaved by the indicated restriction enzymes, in a single ligation reaction. The cloning of the YN1YC155 construct was carried out using the same multiple fragment assembly procedure used in the YN1YC173 construct. For the YN1-154-linker-1 fragment, the gene segment-encoding amino acids 1–154 of eYFP were amplified through PCR using the corresponding primers (forward primer: 5′-CAT ATG GTG AGC AAG GGC GAG GAG-3′; reverse primer: 5′-GGA TCC CAT ATG TGA TGA GGT ACC CAT GAT ATA GAC GTT GTG-3′), which were designed to include the linker-1 sequence (GTSSHM) and desired restriction enzyme cleavage sites (NdeI and BamHI). For the YC155-239-linker-3 fragment, the partial gene that encompassed amino acids 155–239 of eYFP was PCR-amplified

using the designated primers (forward primer: 5′- GTC GAC GCC GAC AAG CAG AAG AAC-3′; reverse primer: 5′- AAG CTT CAT ATG ACC TGA ACC CTT GTA CAG CTC GTC CAT-3′) that incorporated the linker-3 sequence (GSGHM) and the specified restriction enzyme cleavage sites (SalI and HindIII) at the termini. By combining the four PCR fragments (YN1-154-linker-1, GGBP1-32-linker-2, YC155-239-linker-3 and GGBP33-309) and the linearized vector (pET-21a(+)) cleaved by the indicated restriction enzymes, a single ligation reaction was performed, leading to the successful assembly of the YN1YC155 construct. The choice of flexible linkers was based on a previous report that highlighted the flexibility conferred by these small amino acids, enabling the mobility of the connected functional domains [30].
